# An interactive and intuitive visualisation method for X-ray computed tomography data of biological samples in 3D Portable Document Format

**DOI:** 10.1038/s41598-019-51180-2

**Published:** 2019-10-17

**Authors:** Markéta Tesařová, Eglantine Heude, Glenda Comai, Tomáš Zikmund, Markéta Kaucká, Igor Adameyko, Shahragim Tajbakhsh, Jozef Kaiser

**Affiliations:** 1 Central European Institute of Technology, Brno University of Technology, Brno, Czech Republic; 2 Department Adaptation du Vivant, Museum national d’Histoire naturelle, CNRS UMR 7221, Paris, France; 3 Department of Developmental and Stem Cell Biology, Stem Cells and Development Unit, Institut Pasteur, Paris, France; 4 CNRS UMR, 3738 Paris, France; 5 Department of Physiology and Pharmacology, Karolinska Institutet, Solna, Sweden; 6 Department of Molecular Neurosciences, Medical University of Vienna, Vienna, Austria

**Keywords:** Embryology, Biomedical engineering

## Abstract

3D imaging approaches based on X-ray microcomputed tomography (microCT) have become increasingly accessible with advancements in methods, instruments and expertise. The synergy of material and life sciences has impacted biomedical research by proposing new tools for investigation. However, data sharing remains challenging as microCT files are usually in the range of gigabytes and require specific and expensive software for rendering and interpretation. Here, we provide an advanced method for visualisation and interpretation of microCT data with small file formats, readable on all operating systems, using freely available Portable Document Format (PDF) software. Our method is based on the conversion of volumetric data into interactive 3D PDF, allowing rotation, movement, magnification and setting modifications of objects, thus providing an intuitive approach to analyse structures in a 3D context. We describe the complete pipeline from data acquisition, data processing and compression, to 3D PDF formatting on an example of craniofacial anatomical morphology in the mouse embryo. Our procedure is widely applicable in biological research and can be used as a framework to analyse volumetric data from any research field relying on 3D rendering and CT-biomedical imaging.

## Introduction

One of the formidable phenomena in developmental biology is how the shape diversity observed among living organisms is defined and controlled during development and growth. Embryonic patterning is a highly dynamic process implicating multiple molecular mechanisms and cell interactions at the basis of organ formation. In the human embryo, defects in such cellular processes can affect the developmental program leading to congenital disorders. Congenital defects have an incidence of 3% in the human population^1^ and they are causal for up to one-quarter of all reported neonatal deaths^2^. Thus, contextual visualisation of embryonic development is critical to elucidate the origins of malformations.

Multi-disciplinary collectives composed of clinical doctors, biologists, engineers and imaging experts are currently pushing forward the understanding of biological questions using three dimensional (3D) approaches. While the analysis of histological sections remains a mainstay in developmental biology, the reconstruction of 3D volumes from 2D slices has provided important information to understand morphogenesis in mouse and human embryos^3–6^. Non-destructive technologies such as 3D imaging by confocal microscopy and light sheet methods^7–9^, optical projection tomography (OPT)^10–12^ and micro-computed tomography (microCT)^13–16^ constitute emerging powerful methods to analyse the topography of developing structures in 3D context, to gain insight into the pathogenesis of congenital disorders. However, one major challenge of 3D approaches is sharing of complex datasets effectively and intuitively between colleagues from different fields for discussion, and ultimately presenting them in a publication format. Generally, 3D datasets need special software for visualisation or are reduced to 2D pictures in which important information might be lost.

The study of the developing head constitutes a good example of the need for 3D approaches given its complex anatomy. The craniofacial region is built of diverse embryonic cell types giving rise to hard and soft tissues including skeletal, muscular and nervous components^17^. The final shape of the face strongly depends on the geometry of the skeletal elements and their interaction with adjacent soft tissues such as muscles and the nervous system^15,16^. Numerous congenital craniofacial abnormalities affect the form and function of the face and explanations of these malformations still await the fundamental understanding of the underlying failure of morphogenesis^18^. The microCT approach followed by 3D reconstruction has enabled high-quality information of the complex morphological aspects of head and face development^15,16^.

To facilitate assimilation and visualisation of microCT datasets, interactive 3D Portable Document Format (PDF) has been used in different field including in developmental biology^6,19,20^, in human physiology and anatomy^4,21–23^, in entomology^24^ and marine biology^25,26^. However, a major limitation of previously published methods is the requirement of advanced programming skills and/or installation of further prepaid software packages^21^. Most of these approaches depend on the use of Adobe Acrobat Pro Extended software^4,6,19,23–28^ or JavaScript programming language^20,27^.

Here, we provide an alternative, user-friendly way to create interactive 3D PDF files from microCT datasets taking the complexity of mouse craniofacial anatomy as a model example. Our innovative and efficient pipeline comprises microCT data acquisition, segmentation and final establishment of a 3D PDF using a combination of free and pre-paid software. The resulting file can be viewed with standard PDF viewers and offers an interactive interface for microCT data sharing, analysis and presentation.

## Pipeline for the Creation of Interactive 3D PDF

**Figure 1:**
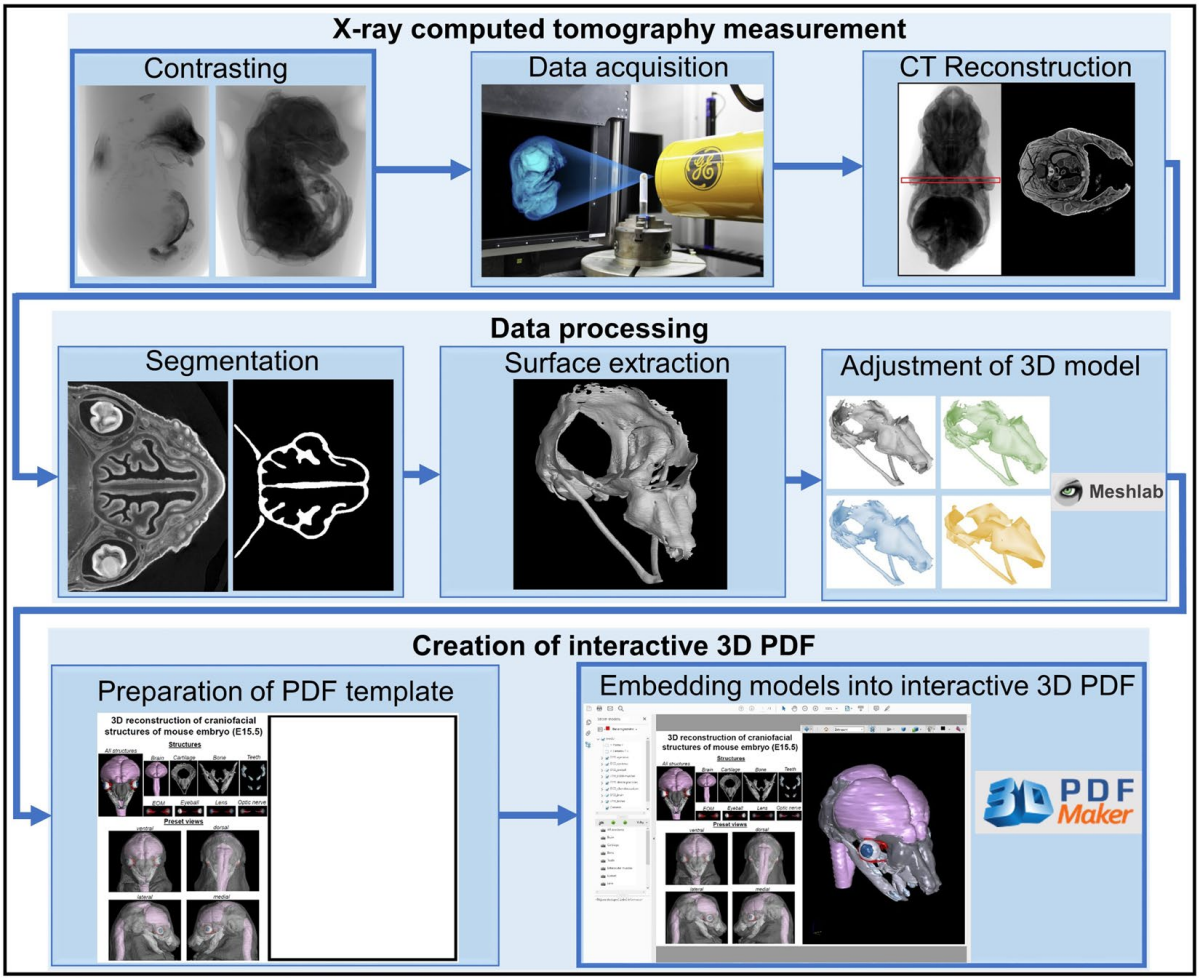
Overview of the method pipeline described in this study.

To create an interactive 3D PDF from microCT data, the critical steps are: (i) chemical contrasting of biological samples if soft tissues are to be visualized, (ii) data acquisition and (iii) CT virtual reconstruction (Fig. 1). This is followed by data processing, the most critical step consisting of segmentation, surface extraction, adjustment of segmented 3D models, and conversion into a final interactive PDF file. Here, we provide a detailed description of each step of the pipeline (see also Supplementary Material 1).

## X-Ray Microtomography Measurement

MicroCT is an established technology for imaging mineralized tissues in animal specimens. However, its use in comparative morphology has been limited by the low intrinsic X-ray contrast of non-mineralised soft tissues. To overcome this problem, methods have been developed to increase tissue contrast, including chemical treatment with contrasting agents^29,30^, phase-contrast imaging^31–36^ or dual-energy computed tomography (DECT)^37^.

**Figure 2:**
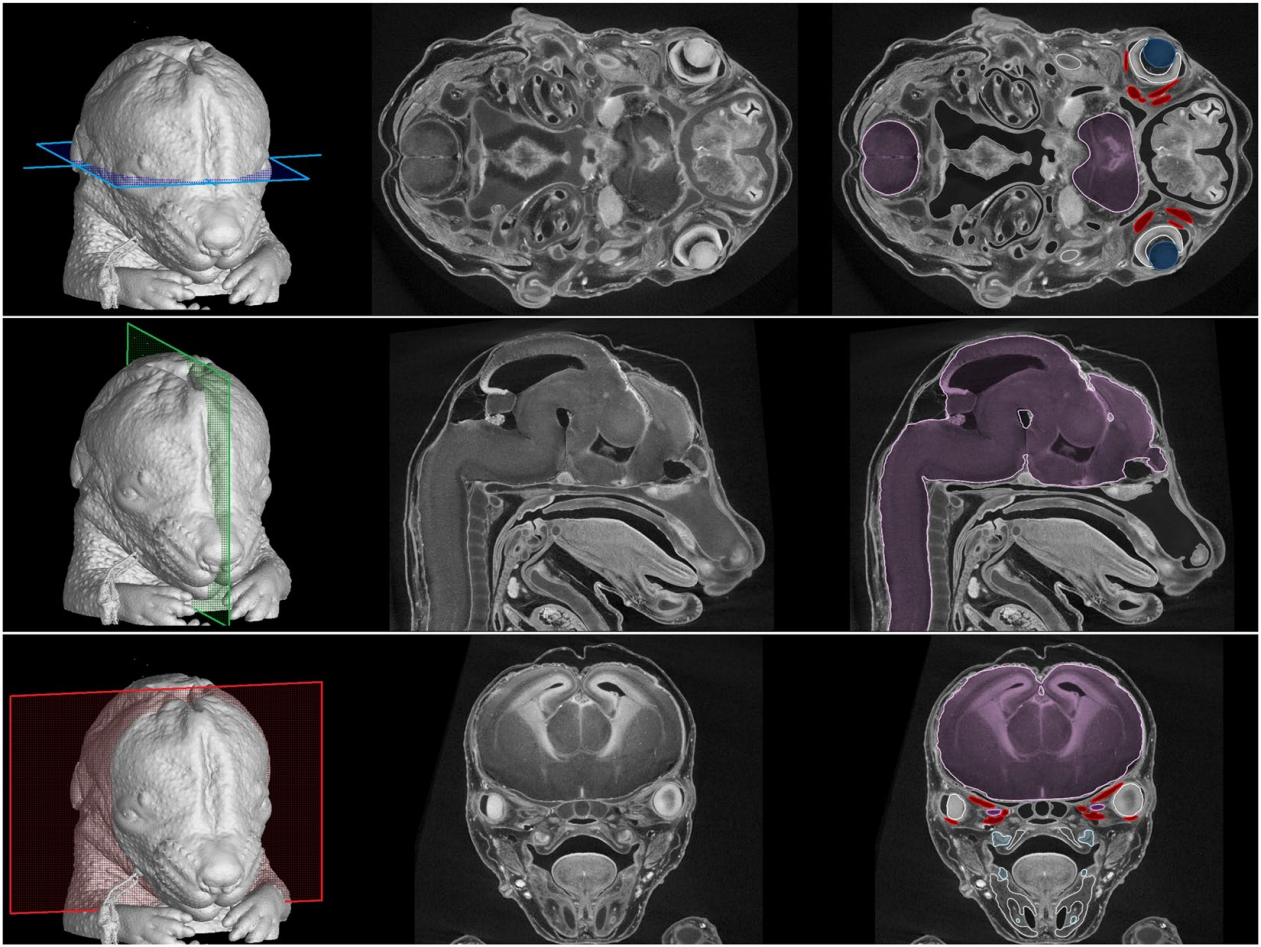
Tomographic measurements and segmentation of craniofacial structures in a mouse embryo 15 days post-fertilisation. Colour planes on 3D models (left panels) indicate the position of the raw tomographic slices (middle panels) and some segmented structures (right panels) including the central nervous system (purple), the lens (dark blue), the dental placodes (light blue) and extraocular muscles (red).

Chemical contrasting treatments have mostly been applied for the characterization of musculoskeletal tissues on small fixed samples. For *in vivo* CT imaging including clinical diagnoses, non-toxic iodine-based contrast agents (e.g. iohexol or hexabrix) are used for the analysis of the cardiovascular system and cavities, but not for direct analysis of muscle tissues^38,39^. Clinical CT imaging of the musculoskeletal system is commonly performed without a contrasting agent, thereby limiting the analysis to discrete soft organs^40^. Therefore, on small fixed samples, multiple contrasting protocols have been used, each with its own advantages and limitations^41,42^. The stainings based on iodine, osmium or the toxic phosphotungstic acid (PTA) are the most commonly used^29,30^. For the pipeline proposed here on the study of craniofacial mouse development, we propose the use of PTA contrasting treatment that permits high contrast imaging of a wide variety of soft and mineralised tissues composing the mouse head (Fig. 2). However, microCT data obtained with other contrasting agents or in their absence, such as clinical data or data from non-biological specimens, will be equally ameneable for the subsequent steps of the 3D PDF pipeline.

## Data Processing

**Figure 3:**
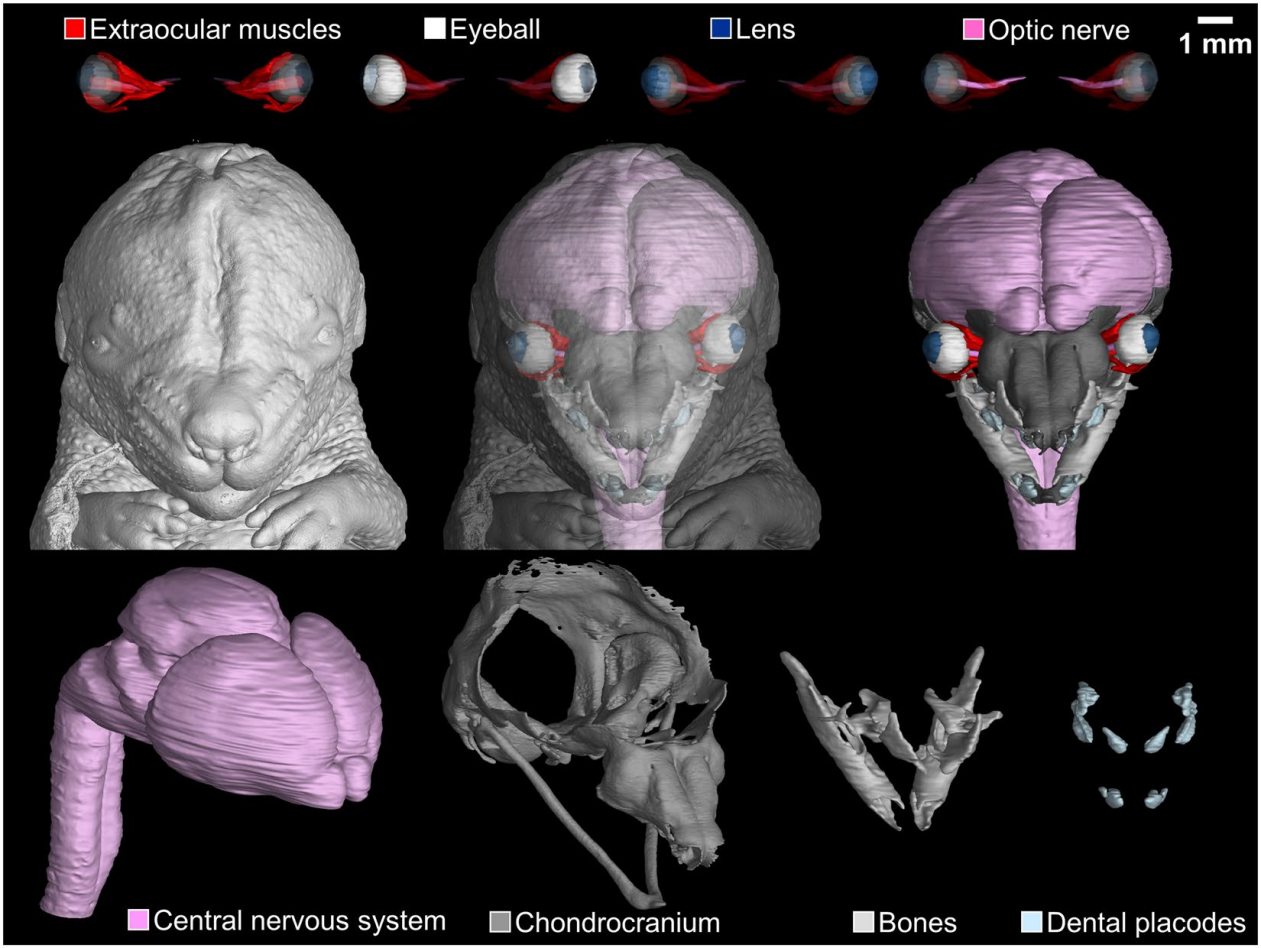
Surface rendering of segmented structures in a mouse embryo 15 days post-fertilisation. Structures of interest are colour-coded.

Following the acquisition of high-contrast tomographic slices, the segmentation of complex craniofacial structures constitutes a major challenge. Fully-automatic approaches have been tried and were generally unsuccessful^43^. Thus, the manual approach is usually the only method available to achieve precise and accurate segmentations^44^. However, some semi-automatic methods such as local segmentation or interpolation between manually segmented slices can be used^45,46^. For verification of the accuracy of manually segmented structures, Fig. 2 shows both original and segmented tomographic slices. This approach permitted the segmentation of soft tissues such as eyeballs, extraocular muscles and the central nervous system, as well as of hard tissues including the chondrocranium, bones and future teeth (dental placodes) (Fig. 3).

For 3D volume rendering, it is then more convenient to use 3D mesh formats rather than a stack of 2D images. 3D mesh formats represent a series of 2D polygons (typically triangles or quadrangles) linked together to recreate the surface of a 3D object^28^. It encodes the 3D model’s geometry, colours, textures, etc. For further work, the segmented masks need to be transferred to the meshes. Most software can transfer the mask into a wide range of equivalent formats. However, only some of the formats contain information about the colour (e.g. OBJ, VRML and STEP) which is important for the visualisation of several structures in one model. Other formats represent only the mesh (e.g. STL and Matlab m-file).

**Figure 4:**
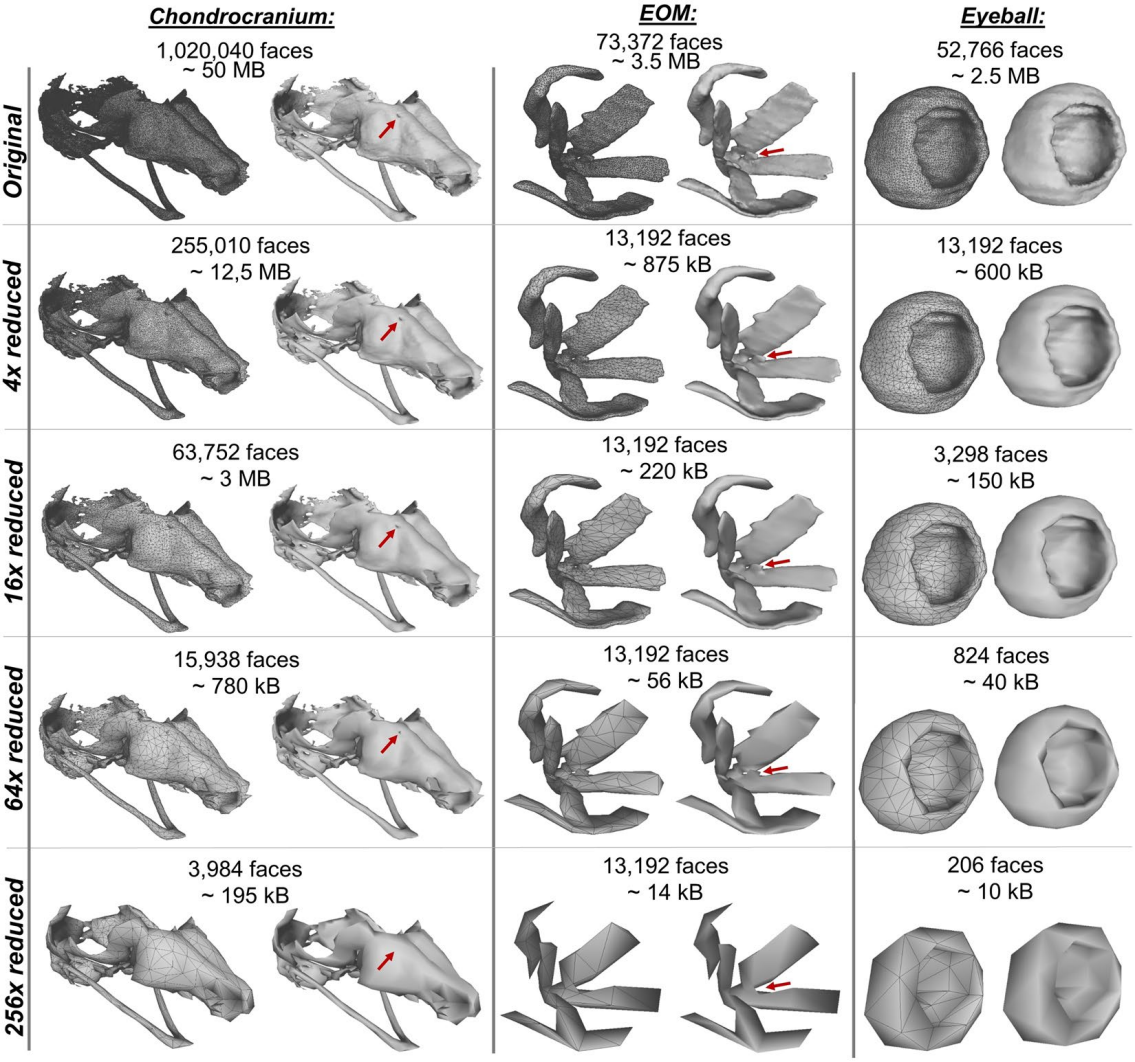
The number of faces affects the detection of details in chondrocranium, extraocular muscles (EOM) and eyeball models. Red arrows indicate details in the 3D model that disappear with model simplification.

Another problem to be solved is the size of the model (i.e. the number of faces in the mesh). When transferring the segmented mask into the mesh, an unnecessarily large number of faces can be created. Comparison of a different number of faces and corresponding size can be found in Fig. 4. Surprisingly, reducing by four times the number of faces does not significantly compromise details in the 3D models. Simple shapes such as the eyeball do not show notable errors besides the slight deformation of the sphere. However, further simplification of complex shapes, such as the chondrocranium and extraocular muscles (EOM), reduces the model quality and some details are lost (Fig. 4, red arrows). Thus, depending on the structures analysed and the resolution needed, different face reductions should be tested and validated before further analysis. Therefore, the compression of the data should be customised according to the complexity of the model and the resolution of tomographic slices.

Among the 3D printing software, some free software enable colour modifications or mesh simplification. Here, we made use of the Meshlab^47^ and Blender^48^ packages for this purpose. Notably, the latter allows unifying different sub-models into one mesh preserving the individuality of each object (see Supplementary Material 1 for more details).

## Creation of an Interactive 3D PDF

Once the models are colour-labelled and simplified, they are then converted into an interactive file. We exploited 3D PDF Maker Standalone^49^ taking advantage of the pre-prepared model that provides an interactive window in the PDF file by clicking on Add 3D button. Before embedding the model into an interactive file, the user can prepare an ordinary PDF file using standard software (e.g. Microsoft Office, Apache OpenOffice etc.). This file is used as a template showing, for example, individual structures or predefined views. These areas (i.e. images or signs) are taken into life in 3D PDF Maker by assigning whatever area in the page to specific view/structure (see for more details Supplementary Materials 1 and 2).

The model document presented here (Supplementary Material 3) consists of four predefined views (ventral, dorsal, lateral and medial) and a possibility of showing individual craniofacial structures (brain, cartilage, bone and teeth). It helps to manipulate the 3D model and can be set according to the areas/views of interest that are important to show. For example, for better visualisation, some structures can be set to the semi-transparent mode, or structures can be switched on/off individually in the model tree. The interactive 3D PDF also allows the user to rotate, turn and pan the model, change the pre-defined 3D-rendering, change the lights and background colour or add a virtual cross-section on the model.

## Discussion and Conclusion

Comparative morphological studies in the field of developmental biology have been challenging, but contrast-enhanced X-ray computed tomography has brought new possibilities of high-resolution 3D visualisation^15,16,50^. In this study, we present a detailed, user-friendly protocol for the surface rendering of craniofacial structures including soft tissues (muscles, eyeballs, central nervous system) as well as of hard tissues (cartilages, bones and teeth), with a step-by-step procedure from sample collection to the creation of an interactive 3D PDF. The final PDF is readable on all operating systems with the free standard AdobeReader®/AcrobatReaderDC (Adobe Inc., California USA).

Our 3D reconstruction methodology has already shown its utility and strength for visualisation and phenotypic analysis of complex structures such as the neck musculoskeletal system^50^ and nasal capsules of control and mutant mouse embryos^16^. Therefore, a user-friendly method for creating such files will be of great utility for biologists. In addition, our procedure can be used as a framework to analyse volumetric data for any field of research that relies on 3D rendering, e.g. for visualisation of volumetric information of geological samples by laser-induced breakdown spectroscopy^51^.

The use of interactive 3D PDF files has a great potential for data sharing, communication and publications^21^, but is still sparsely used. We believe that our work will inspire researchers working with 3D imaging to present their data in such a user-friendly format.

## Methods

### Use of experimental animals

All animal work was approved and permitted by the Local Ethical Committee on Animal Experiments (North Stockholm Animal Ethics Committee) and conducted according to The Swedish Animal Agency’s Provisions and Guidelines for Animal Experimentation recommendations. Mice were sacrificed with isoflurane (Baxter KDG9623) overdose or cervical dislocation, and embryos were collected into ice-cold PBS. Subsequently, tissue samples were fixed into freshly prepared 4% paraformaldehyde (PFA) in PBS solution for 24 hours at +4 °C with slow rotation and washed in PBS.

### Tissue contrasting

Staining protocol has been adapted and modified from the original protocol developed by Brian Metscher laboratory^33,34^. After fixation, the samples were dehydrated in increasing concentration of ethanol series (30%, 50%, 70%, 90%), one day in each concentration to minimise the shrinkage of tissues. For tissue contrasting, samples were then transferred into 1% PTA (phosphotungstic acid) in 90% methanol for three weeks. The PTA-methanol solution was changed every two days. Subsequently, the samples were rehydrated by ethanol series (90%, 70%, 50% and 30%) and shipped to the CT-laboratory for scanning.

### X-ray microCT measurements

After fixation and contrasting treatments, samples were fully rehydrated in distilled water, embedded in 1.0% agarose gel and placed in a polypropylene tube to avoid movement artefacts during tomography scanning. The polypropylene tube was fixed on a plastic rod by a silicone gun. The rod was put in the centre of the rotation stage axis. The microCT scanning was performed using the laboratory system GE Phoenix v|tome|x L 240 (GE Sensing & Inspection Technologies GmbH, Germany) with a 180 kV/15 W maximum power nanofocus X-ray tube and flat panel dynamic 41|100 with 4000 × 4000 px and a pixel size of 100 × 100 μm. The exposure time was 600 ms in each of the 2200 projections acquired over a total angle of 360°. Three projections were acquired and averaged in every position to reduce the noise in the tomographic data. Thus, the scanning time was 73 minutes. The acceleration voltage and current of the X-ray tube were 60 kV and 200 μA, respectively. The beam was filtered by a 0.2 mm-thick aluminium filter. The linear voxel resolution of the measurement was set to 4.2 μm in all dimensions. The tomographic reconstruction was conducted using the software GE phoenix datos|x 2.0 (GE Sensing & Inspection Technologies GmbH, Germany).

### Data processing and analysis

Segmentation of craniofacial structures was performed semi-manually in Avizo (Thermo Fisher Scientific, USA). We used some automatic tools using a region growing method and thresholding on 2D slices. Segmentations were done on one of three to five slices, and the mask was interpolated. Subsequently, the segmented models were smoothed in VG Studio MAX 3.2 (Volume Graphics GmbH, Germany) according to^45^. The segmented mask was then exported into a mesh (*.OBJ format) and was colour-labelled and adjusted in Meshlab^47^ and Blender software^48^. The colour-coded models were transferred into a pre-prepared PDF file in 3D PDF Maker Standalone^49^. The detailed manual can be found in Supplementary Material 1. The use of the interactive 3D PDF is available in Supplementary Material 2.

## Supplementary information

Supplementary information

Supplementary Dataset 1

Supplementary Dataset 2

Supplementary Dataset 3

## Data Availability

The datasets generated during and/or analysed during the current study are available from the corresponding author on reasonable request.
