# Supplementary material for: An interactive and intuitive visualisation method for X-ray computed tomography data of biological samples in 3D Portable Document Format
Source: Sci Rep. 2019 Oct 17;9:14896. doi: 10.1038/s41598-019-51180-2 (PMC6797759; doi:10.1038/s41598-019-51180-2)
Supplement: Supplementary file 1 — Supplementary information [file 41598_2019_51180_MOESM1_ESM.docx]

**An interactive and intuitive visualisation method for X-ray computed tomography data of biological samples in 3D Portable Document Format**

Markéta Tesařová^1^, Eglantine Heude^2,3,4^, Glenda Comai^3,4^, Tomáš Zikmund^1^, Markéta Kaucká^5,6^, Igor Adameyko^5,6^, Shahragim Tajbakhsh^3,4^ and Jozef Kaiser^1*^

^1^Central European Institute of Technology, Brno University of Technology, Brno, Czech Republic

^2^ Department Adaptation du Vivant, Museum national d’Histoire naturelle, Paris, France

^3^ Department of Developmental and Stem Cell Biology, Stem Cells and Development Unit, Institut Pasteur, Paris, France

^4^ CNRS UMR 3738, Paris, France

^5^ Department of Physiology and Pharmacology, Karolinska Institutet, Solna, Sweden

^6^ Department of Molecular Neurosciences, Medical University of Vienna, Vienna, Austria

*Corresponding author: [kaiser@fme.vutbr.cz](mailto:kaiser@fme.vutbr.cz)

**Supplementary information**

Supplementary material 1 – Manual for creation of 3D PDF.

Supplementary material 2 – Information on the use of the interactive 3D PDF.

Supplementary material 3 – Interactive 3D PDF file: 3D reconstruction of craniofacial structures of mouse embryo (E15.5)
