# Supplementary material for: An interactive and intuitive visualisation method for X-ray computed tomography data of biological samples in 3D Portable Document Format
Source: Sci Rep. 2019 Oct 17;9:14896. doi: 10.1038/s41598-019-51180-2 (PMC6797759; doi:10.1038/s41598-019-51180-2)
Supplement: Supplementary file 2 — Supplementary Dataset 1 [file 41598_2019_51180_MOESM2_ESM.pdf]

An interactive and intuitive visualisation method for X-ray computed tomography data of biological samples in 3D Portable Document Format

Markéta Tesařová[1], Eglantine Heude[2,3,4], Glenda Comai[3,4], Tomáš Zikmund[1], Markéta Kaucká[5,6], Igor Adameyko[5,6], Shahragim Tajbakhsh[3,4] and Jozef Kaiser[1*]

[1]Central European Institute of Technology, Brno University of Technology, Brno, Czech Republic
[2] Department Adaptation du Vivant, Museum national d'Histoire naturelle, Paris, France
[3] Department of Developmental and Stem Cell Biology, Stem Cells and Development Unit, Institut Pasteur, Paris, France
[4] CNRS UMR 3738, Paris, France
[5] Department of Physiology and Pharmacology, Karolinska Institutet, Solna, Sweden
[6] Department of Molecular Neurosciences, Medical University of Vienna, Vienna, Austria
*Corresponding author: kaiser@fme.vutbr.cz

Supplementary Material 1
Manual for creation of 3D PDF

The manual contains a detailed description of the pipeline for the creation of a personalised interactive 3D PDF file. Our procedure involves a combination of free (Meshlab, Blender) and commercial (Avizo, VG Studio, 3D PDF Maker) software. Alternatively, segmentation or remeshing of the model can be done with other appropriate software.

1a. Segmentation (Avizo)

- ✓ Import image stack into Avizo software (DICOM, tiff, raw etc.)
  Click on: *Open data* - mark all images in the stack - option *Read complete volume into memory*.
- ✓ The loaded volume of data appears in a file format *samples_name.vol*.
  Select this file for all processes with volumetric data.
- ✓ Open segmentation editor: Right click on file *samples_name.vol* - *Image segmentation* -option *Edit new label field*.
  Each label field represents one object (anatomical structure) or a type of objects (e.g. dental placodes, muscles etc.)
- ✓ Adjust the contrast by changing the histogram in the right panel.
- ✓ The segmentation is performed using the following software tools:

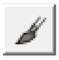

▪ Brush—manual drawing; Size of the brush can be changed according to the dimensions of the segmented object

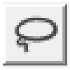

▪ 2D lasso—useful for filling borders of larger, in homogeneous areas.

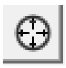

▪ Blow tool—uses region growing method for areas with distinct borders and homogenous filling inside. The segmented area spreads until it reaches a threshold represented by a grayscale value.

- ✓ Perform manual segmentation for approximately one from three to ten slices (depending on object heterogeneity).
  Compute the slice gaps by interpolation (*ctrl + I*) and check the interpolated slices. This method can lead to incompleteness that should be corrected manually.
- ✓ After interpolation, click on *Add selected voxels*.
- ✓ To visualise the segmented area, move back to project scene tree and mark item *Labels – Display - Isosurface - Apply*.
- ✓ To export segmented mask into the mesh, mark selected item of labels - *Compute - Generate Surface – Apply*. The computed surface appears in the right panel as file *samples_name.surf*.

- ✓ Export the mesh by marking item file *samples_name.surf* - click on *Save Data As* - select any of formats (STL ascii, STL little endian etc.)

1b. Smoothing of the model (VG Studio) *(Optional)*

- ✓ Import image stack into the VG Studio software.
  Click on: *File* - *Import – Image stack/Raw volume/DICOM image stack ...*
- ✓ Import mesh to VG studio software into raw data. Click on: *File* - *Import – Mesh.*
- ✓ Transfer mesh to the region of interest in the volumetric data — software function *ROI from the mesh* in the left panel:

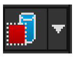

- ✓ Use the function *Smoothing* (left panel) if distinct inaccuracies occurred during interpolation of manual segmentation:

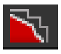

- ✓ Right click on the smoothened region of interest – *Convert to mesh* – click on *Export – Convert.*

2. Remeshing and colour-labelling of the model (Meshlab)

- ✓ Import the meshes – take your mesh from the folder and drop it in the scene.
- ✓ Window with a sign *Unify duplicates* appears – click on *OK.*
- ✓ All meshes appear in the right panel. Change visibility by clicking on/off :

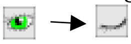

- ✓ When adjusting the model, select the appropriate mesh in the right panel.
- ✓ To visualise detailed structures (faces) of the mesh, click on W*ireframe* icon 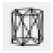 in the top panel.
- ✓ To reduce the size of the model (i.e. the number of faces), in top panel click on: *Filters – Remeshing simplification and reconstruction – Simplification: quadric edge collapse* (or choose other appropriate options for model simplification).
  In the new window, determine *Percentage reduction* (0.5 = reduction of 50%).
  Repeat this step if needed. The simplified model can be observed in the window in real time. The appropriate size of a final model is usually between 1-20 MB.
- ✓ Repeat these steps for all meshes.
- ✓ For complete colour-labelling of the model, colour-labelling of vertex and faces is required:
  - For vertex colour-labelling, click on: *Filters – colour creation and processing – perlin colour* (or choose other appropriate options).
  - For faces colour-labelling, click on: *Filters – colour creation and processing – transfer colour vertex to face – apply*.
- ✓ Once the models are remeshed and colour-labelled, export new meshes:
  In the right panel, click on the mesh to be exported.
  In the top panel, click on *Export mesh as* (choose the format that can contain information about the colour e.g. OBJ, VRML and STEP) – *OK*. Do not use diacritics or space in the model name.

3. Creation of mesh composed of sub-meshes (Blender)

- ✓ Import mesh into Blender software, click on: *File – Import – OBJ* and select the mesh.
- ✓ Repeat this step for all meshes that will be merged into one 3D structure.
- ✓ Export final mesh, click on *Export as wavefront – OBJ.*
  - All meshes that have been imported individually will be exported altogether as one OBJ file, but keeping their individuality.

4. Pre-preparing of the PDF template (Powerpoint) *(Optional)*

- ✓ Before creating an interactive file, you can prepare an ordinary PDF file from Powerpoint, which is used as a template for future 3D PDF. It can shows e.g. signs, individual structures, predefined views, etc.

5. Creation of interactive 3D PDF (3D PDF Maker)

- ✓ Launch 3D PDF Maker Software. Then, you can choose between two options:
  - Open pre-prepared PDF template with static images.
  - Start with a new blank page.
- ✓ To add a 3D model into the page, click on *Add 3D* button 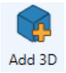

 and select the mesh to be inserted.
- ✓ You can predefine the default view and background colour in this step (that can also be modified later). Then select an area for an interactive window by dragging the mouse.
- ✓ All options regarding the interactive window are set in the top panel by button *Edit selected 3D element*: 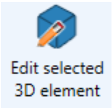

- ✓ By activating this button, you can:
  - Change default view, background colour, lighting scheme and rendering style:

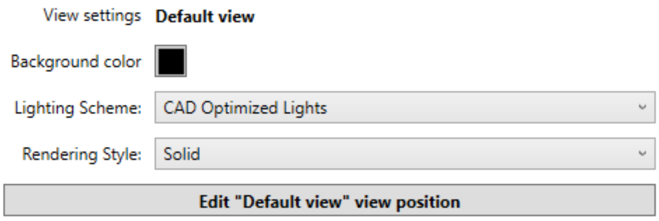

  - Add new views:

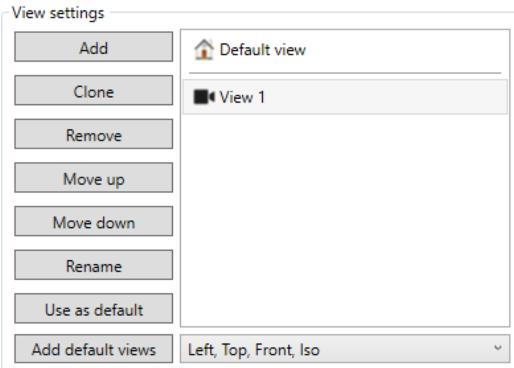

  - Change transparency of objects:

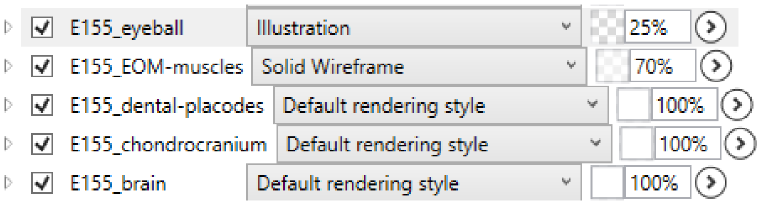

- ✓ Linking pre-defined view with some image/sign can be done by clicking on *Add link to 3D view* button (this function allows the user to create interactive areas in the final PDF).

Drag the mouse in the area that you want to make interactive. The list with predefined views will appear, choose the view that you want to associate with this area.

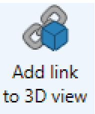

- ✓ You can check the provisional form of the PDF by *Show preview with Adobe Reader* button:

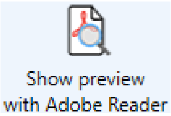

- ✓ To save the final version, just click on the *Save icon*: 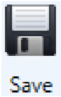

Note: A free 30-day trial to test the 3D PDF Maker software is available on www.3dpdfmaker.com.
