# Supplementary material for: An interactive and intuitive visualisation method for X-ray computed tomography data of biological samples in 3D Portable Document Format
Source: Sci Rep. 2019 Oct 17;9:14896. doi: 10.1038/s41598-019-51180-2 (PMC6797759; doi:10.1038/s41598-019-51180-2)
Supplement: Supplementary file 3 — Supplementary Dataset 2 [file 41598_2019_51180_MOESM3_ESM.pdf]

An interactive and intuitive visualisation method for X-ray computed tomography data of biological samples in 3D Portable Document Format

Markéta Tesařová[1], Eglantine Heude[2,3,4], Glenda Comai[3,4], Tomáš Zikmund[1], Markéta Kaucká[5,6], Igor Adameyko[5,6], Shahragim Tajbakhsh[3,4] and Jozef Kaiser[1*]

[1]Central European Institute of Technology, Brno University of Technology, Brno, Czech Republic
[2] Department Adaptation du Vivant, Museum national d'Histoire naturelle, Paris, France
[3] Department of Developmental and Stem Cell Biology, Stem Cells and Development Unit, Institut Pasteur, Paris, France
[4] CNRS UMR 3738, Paris, France
[5] Department of Physiology and Pharmacology, Karolinska Institutet, Solna, Sweden
[6] Department of Molecular Neurosciences, Medical University of Vienna, Vienna, Austria
*Corresponding author: kaiser@fme.vutbr.cz

Supplementary Material 2
Information on the use of the interactive 3D PDF

- ✓ To view interactive PDF files, use the free standard AdobeReader®/AcrobatReaderDC (www.adobe.com/downloads/). If you see a warning message in the yellow dialogue box on the file opening, you can activate the content by pressing the button "Options", otherwise you can enable 3D data for all documents in the preferences dialogue box (Edit / Preferences / 3D&Multimedia / check the box "Enable playing of 3Dcontent".)
- ✓ In the left part of the document, you can show/hide a *Model tree* of the 3D model, where you can activate/deactivate individual structures:

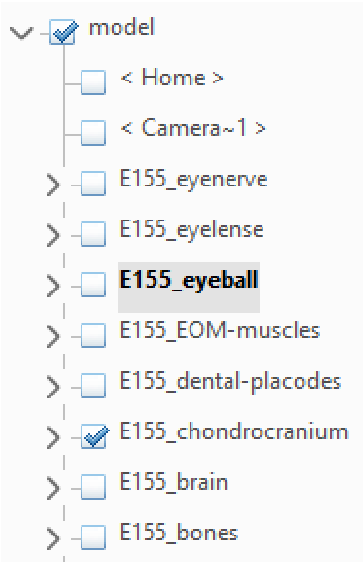

✓ By putting the mouse cursor over the interactive window, a menu with additional options will appear:

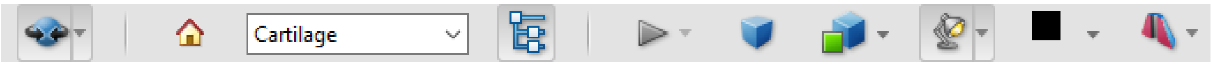

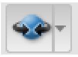 Selection of interaction with the model (rotate, turn, pan, zoom etc.) A possibility of 3D measurement tool …

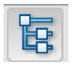 Show/hide the model tree.

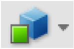 Change of pre-defined 3D rendering.

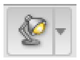 Change of pre-defined lights.

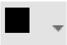 Change of pre-defined background colour.

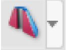 Virtual cross-section on the model.
