# Supplementary material for: An interactive and intuitive visualisation method for X-ray computed tomography data of biological samples in 3D Portable Document Format
Source: Sci Rep. 2019 Oct 17;9:14896. doi: 10.1038/s41598-019-51180-2 (PMC6797759; doi:10.1038/s41598-019-51180-2)

An interactive and intuitive visualisation method for X-ray computed tomography data of biological samples in 3D Portable Document Format

Markéta Tesařová[1], Eglantine Heude[2,3,4], Glenda Comai[3,4], Tomáš Zikmund[1], Markéta Kaucká[5,6], Igor Adameyko[5,6], Shahragim Tajbakhsh[3,4] and Jozef Kaiser[1*]

[1]Central European Institute of Technology, Brno University of Technology, Brno, Czech Republic
[2] Department Adaptation du Vivant, Museum national d'Histoire naturelle, Paris, France
[3] Department of Developmental and Stem Cell Biology, Stem Cells and Development Unit, Institut Pasteur, Paris, France
[4] CNRS UMR 3738, Paris, France
[5] Department of Physiology and Pharmacology, Karolinska Institutet, Solna, Sweden
[6] Department of Molecular Neurosciences, Medical University of Vienna, Vienna, Austria
*Corresponding author: kaiser@fme.vutbr.cz

3D reconstruction of craniofacial structures of mouse embryo (E15.5)

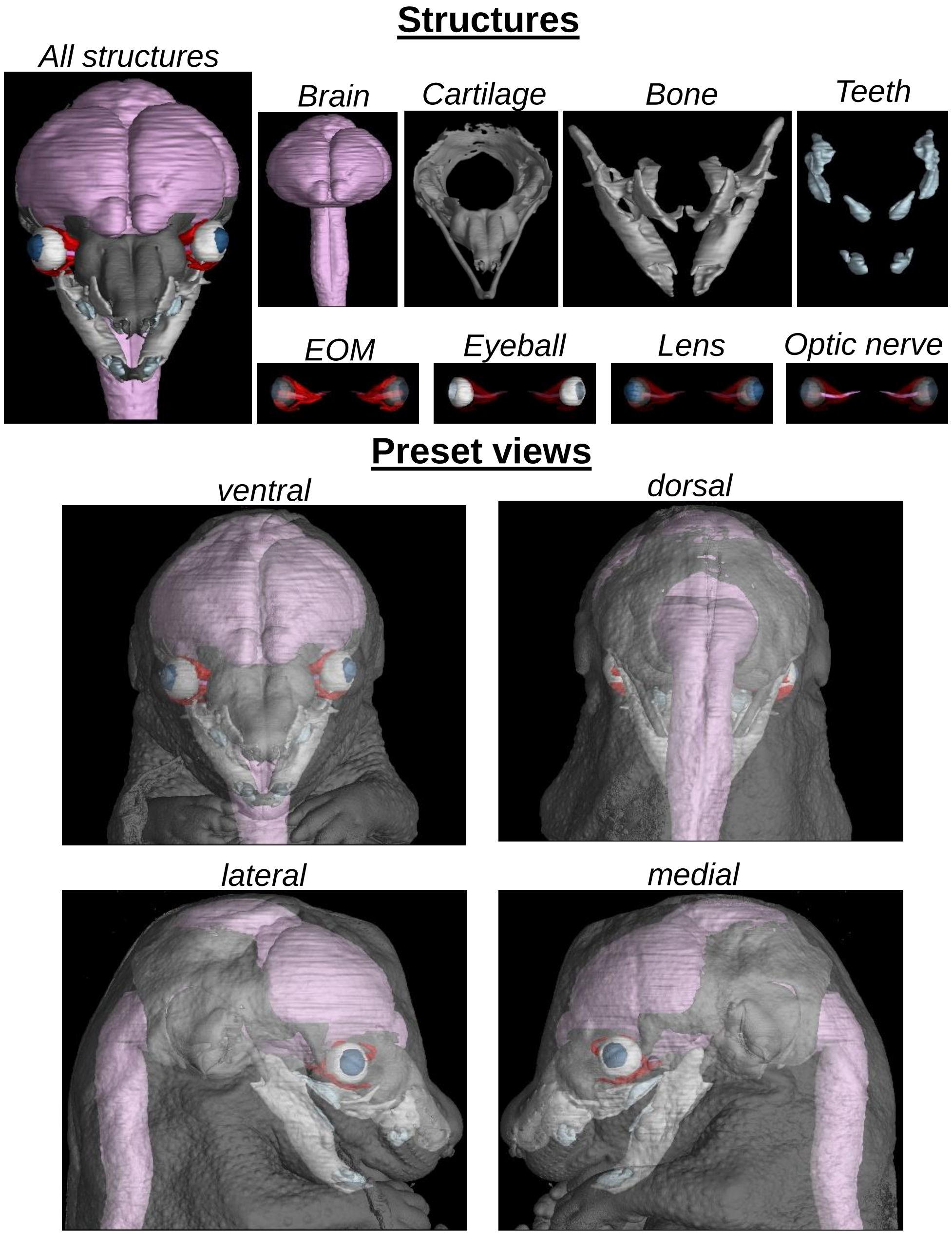

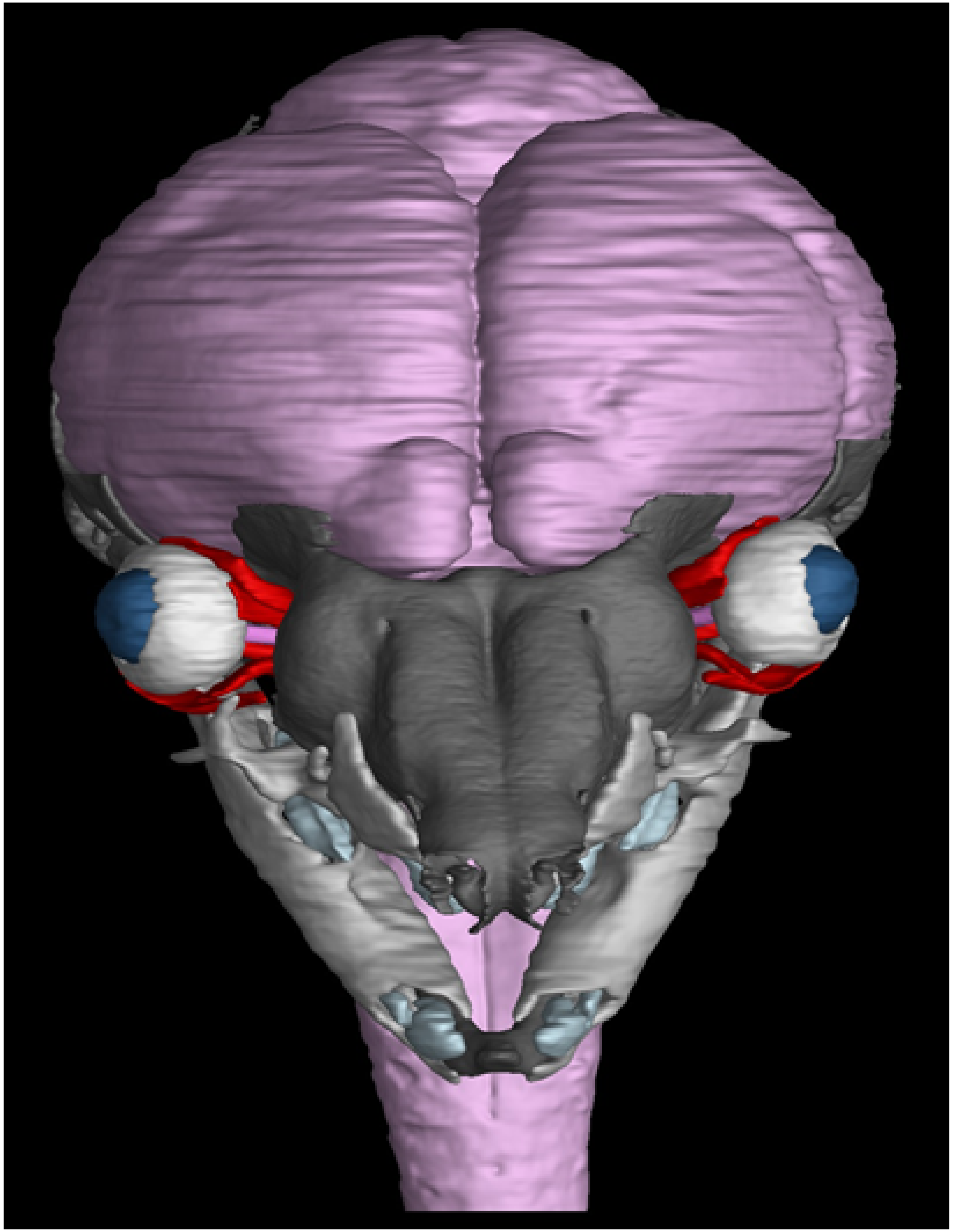

Supplement: Supplementary file 4 — Supplementary Dataset 3 [file 41598_2019_51180_MOESM4_ESM.pdf]
